# Zerumbone Suppresses the LPS-Induced Inflammatory Response and Represses Activation of the NLRP3 Inflammasome in Macrophages

**DOI:** 10.3389/fphar.2021.652860

**Published:** 2021-05-11

**Authors:** Chia-Cheng Su, Shu-Chi Wang, I-Chen Chen, Fang-Yen Chiu, Po-Len Liu, Chi-Han Huang, Kuan-Hua Huang, Shih-Hua Fang, Wei-Chung Cheng, Shu-Pin Huang, Hsin-Chih Yeh, Ching-Chih Liu, Po-Yen Lee, Ming-Yii Huang, Chia-Yang Li

**Affiliations:** ^1^ Graduate Institute of Medicine, College of Medicine, Kaohsiung Medical University, Kaohsiung, Taiwan; ^2^ Division of Urology, Department of Surgery, Chi-Mei Medical Center, Tainan, Taiwan; ^3^ Department of Senior Citizen Service Management, Chia Nan University of Pharmacy and Science, Tainan, Taiwan; ^4^ Department of Medical Laboratory Science and Biotechnology, Kaohsiung Medical University, Kaohsiung, Taiwan; ^5^ Department of Pediatrics, Kaohsiung Medical University Hospital, Kaohsiung, Taiwan; ^6^ Department of Pediatrics, School of Medicine, College of Medicine, Kaohsiung Medical University, Kaohsiung, Taiwan; ^7^ Department of Respiratory Therapy, College of Medicine, Kaohsiung Medical University, Kaohsiung, Taiwan; ^8^ Institute of Athletics, National Taiwan University of Sport, Taichung, Taiwan; ^9^ Graduate Institute of Biomedical Science, Research Center for Cancer Biology, China Medical University, Taichung, Taiwan; ^10^ Center for Cancer Research, Kaohsiung Medical University, Kaohsiung, Taiwan; ^11^ Department of Urology, School of Medicine, College of Medicine, Kaohsiung Medical University, Kaohsiung, Taiwan; ^12^ Department of Urology, Kaohsiung Medical University Hospital, Kaohsiung Medical University, Kaohsiung, Taiwan; ^13^ Department of Urology, Kaohsiung Municipal Ta-Tung Hospital, Kaohsiung, Taiwan; ^14^ Department of Ophthalmology, Chi Mei Medical Center, Taichung, Taiwan; ^15^ Department of Ophthalmology, Kaohsiung Medical University Hospital, Kaohsiung Medical University, Kaohsiung, Taiwan; ^16^ Department of Radiation Oncology, Cancer Center, Kaohsiung Medical University Hospital, Kaohsiung Medical University, Kaohsiung, Taiwan; ^17^ Department of Medical Research, Kaohsiung Medical University Hospital, Kaohsiung, Taiwan

**Keywords:** zerumbone, macrophage, inflammation, NLRP3 inflammasome, MAPKs

## Abstract

Zerumbone is a natural product isolated from the pinecone or shampoo ginger, *Zingiber zerumbet* (L.) Smith, which has a wide range of pharmacological activities, including anti-inflammatory effects. However, the effects of zerumbone on activation of the NLRP3 inflammasome in macrophages have not been examined. This study aimed to examine the effects of zerumbone on LPS-induced inflammatory responses and NLRP3 inflammasome activation using murine J774A.1 cells, murine peritoneal macrophages, and murine bone marrow-derived macrophages. Cells were treated with zerumbone following LPS or LPS/ATP treatment. Production of nitric oxide (NO) was measured by Griess reagent assay. The levels of IL-6, TNF-α, and IL-1β secretion were analyzed by ELISA. Western blotting analysis was performed to determine the expression of inducible NO synthase (iNOS), COX-2, MAPKs, and NLRP3 inflammasome-associated proteins. The activity of NF-κB was determined by a promoter reporter assay. The assembly of NLRP3 was examined by immunofluorescence staining and observed by confocal laser microscopy. Our experimental results indicated that zerumbone inhibited the production of NO, PGE_2_ and IL-6, suppressed the expression of iNOS and COX-2, repressed the phosphorylation of ERK, and decreased the activity of NF-κB in LPS-activated J774A.1 cells. In addition, zerumbone suppressed the production of IL-1β and inhibited the activity of NLRP3 inflammasome in LPS/ATP- and LPS/nigericin-activated J774A.1 cells. On the other hand, we also found that zerumbone repressed the production of NO and proinflammatory cytokines in LPS-activated murine peritoneal macrophages and bone marrow-derived macrophages. In conclusion, our experimental results demonstrate that zerumbone effectively attenuates the LPS-induced inflammatory response in macrophages both *in vitro* and *ex vivo* by suppressing the activation of the ERK-MAPK and NF-κB signaling pathways as well as blocking the activation of the NLRP3 inflammasome. These results imply that zerumbone may be beneficial for treating sepsis and inflammasome-related diseases.

## Introduction

Inflammasomes are multi-protein complexes mainly located in macrophages, dendritic cells, and some other immune cells, which mediate activation of the proteolytic enzyme caspase-1 and subsequently regulate the secretion of the proinflammatory cytokines, interleukin (IL)-1β and IL-18, as well as actuating pyroptosis, a form of cell death induced by bacterial pathogens (Franchi et al., 2009). One of the most intensively studied inflammasomes is the NLR family pyrin domain containing 3 (NLRP3) inflammasomes, consisting of the sensor molecule NLRP3 protein, the adaptor protein apoptosis-associated speck-like protein containing a CARD (ASC), and procaspase-1 (Kelley et al., 2019). Dysregulation of the NLRP3 inflammasome activation has been demonstrated to associate with development and progression of various diseases including Alzheimer’s disease (Tan et al., 2013), sepsis (Danielski et al., 2020), gouty (Yang et al., 2018), Inflammatory Bowel disease (Mao et al., 2018), and systemic lupus erythematosus (Tan et al., 2019), etc. Hence, the NLRP3 inflammasome has been considered a promising target for the treatment of many diseases associated with inflammation.

The activation of the NLRP3 inflammasome requires a two-step process: a priming step and a subsequent activation step. The priming step is provided by inflammatory stimuli such as Toll-like receptor 4 (TLR4) agonists (ex. lipopolysaccharide, LPS) that induce mitogen-activated protein kinase (MAPK) signaling [e.g., the extracellular signal-regulated kinases (ERK), the c-Jun N-terminal kinases (JNK), and the p38 MAPKs (p38)] and the nuclear factor-κB (NF-κB)-mediated NLRP3 and pro-IL-1β expression (Yang et al., 2019). The subsequent activation step leads to NLRP3 inflammasome oligomerization and triggers the assembly of a multi-protein complex comprised of NLRP3, ASC, and procaspase-1. Three main activating mechanisms have been proposed to induce the activation of subsequent activation steps: K^+^ efflux, mitochondrial dysfunction and generation of mitochondria-derived reactive oxygen species (ROS), and phagolysosomal destabilization in response to particulates (Ratsimandresy et al., 2013).

*Zingiber zerumbet* (L.) Smith, commonly known as the pinecone or shampoo ginger, has long been used as a botanical medicine in Asia and India, and the rhizomes of *Z. zerumbet* have been traditionally used for the management of numerous inflammatory disorders including allergies, fever, asthma, severe sprains, torment, toothache, stomachache, and wounds (Yob et al., 2011; Haque et al., 2018). Zerumbone is a main component isolated from *Zingiber zerumbet* (L.) Smith, which has been studied for its effectiveness in various biological activities such as antiulcer (Al-Amin et al., 2012), antioxidant (Rosa et al., 2019), anticancer (Girisa et al., 2019), and antimicrobial (Shin and Eom, 2019). A previous study indicated that zerumbone suppresses the activation of inflammatory mediators in LPS-stimulated U937 macrophages through MyD88-dependent NF-κB/MAPK/PI3K-Akt signaling pathways (Haque et al., 2018). In addition, zerumbone has been reported to suppress the regulation of pro-inflammatory responses on LPS-activated inflammation of THP-1 cell-derived macrophages (Kim and Yun, 2019). However, the effect of zerumbone on the regulation of NLRP3 inflammasome activation on macrophages has not been explored. Therefore, the aim of this study was to examine the effect of zerumbone in regulating LPS-induced inflammatory responses and NLRP3 inflammasome activation in macrophages.

## Materials and Methods

### Ethics Statement

All animal handling and procedures were approved by the Committee on the Ethics of Animal Experiments of the Kaohsiung Medical University (Permit Number: 104092). All animal works were performed in an Association for Assessment and Accreditation of Laboratory Animal Care International (AAALAC)-accredited facility.

### Reagents

Zerumbone (purity ≧ 98%) was purchased from ChemFaces (Wuhan, Hubei, China), and the results of the limulus amebocyte lysate single test (Associates of Cape Cod, Inc. Falmouth, MA, United States) indicated that endotoxin level of zerumbone was lower than the detection limit (less than 0.03 EU/mL). RPMI-1640 medium, fetal bovine serum (FBS), penicillin, streptomycin, phosphate buffered saline (PBS) were purchased from Corning Cellgro, Inc (Corning, NY, United States). LPS (from *E. coli* O111:B4), 3 - (4,5-dimethylthiazol-2-yl)-2, 5-diphenyl tetrazolium bromide (MTT), adenosine 5′-triphosphate (ATP), nigericin, Tween-20, and DMSO were purchased from Sigma Aldrich (St. Louis, MO, United States). For the Western blot, primary antibodies against iNOS (inducible nitric oxide synthase, sc-651), COX-2 (cyclooxygenase 2, sc-166475), ASC (sc-22514-R), and IL-1β (sc-7884) were purchased from Santa Cruz Biotechnology (Santa Cruz, CA, United States); phospho-ERK1/2 (CST#4370), ERK1/2 (CST#4695), phospho-JNK1/2 (CST#9251), JNK1/2 (CST#9258), phospho-p38 MAPK (CST#4511), p38 MAPK (CST#8690), cleaved caspase-1 (CST#67314), cleaved IL-1β (CST#52718) were obtained from Cell Signaling (Farmingdale, NY, United States); caspase-1 (AG-20B-0042) was obtained from AdipoGen (San Diego, CA, United States); β-actin (MA5-15739) was purchased from Thermo Fisher Scientific (Waltham, MA, United States). For the treatment of zerumbone, zerumbone was dissolved in DMSO at a stock concentration of 50 mM, then further diluted in the culture medium at a final DMSO concentration of ≤0.02%.

### Cell Culture

Murine macrophage J774A.1 cells were purchased from Bioresource Collection and Research Center (Food Industry Research and Development Institute, Hsinchu, Taiwan). Mouse L929 fibroblasts were purchased from American Type Culture Collection (Manassas, MD, United States). Cells were cultured in RPMI-1640 medium supplemented with 10% FBS and antibiotics (100 U/mL penicillin and 100 U/mL streptomycin) and passaged every 2–3 days to maintain growth at 37°C in a humidified incubator containing 5% CO_2_.

### Isolation of Peritoneal Macrophages and Bone Marrow-Derived Macrophages

For the isolation of murine peritoneal macrophages, the specific pathogen-free female C57BL/6 mice [6–8 weeks of age, female mice have greater inflammatory response than male mice (Klein and Flanagan, 2016)] were intraperitoneally injected with 1 ml sterile 3% thioglycolate medium and peritoneal macrophages were collected after 4 days, as described previously (Hung et al., 2019). Murine bone marrow cells were isolated from the femur according to a previous study (Liu and Quan, 2015), and cells were then cultured in RPMI 1640 medium containing 10% FBS and 15% L929 cell-conditioned medium (LCM) for seven days for macrophage differentiation following previous studies (Trouplin et al., 2013; Tang et al., 2014). The LCM contained a granulocyte-macrophage colony-stimulating factor (GM-CSF) that is a lineage-specific growth factor to induce the proliferation and differentiation of committed myeloid progenitors into cells of the macrophage/monocyte lineage (Trouplin et al., 2013; Tang et al., 2014).

### Cytotoxicity Assay

Cell viability was determined using MTT assay as described previously (Hung et al., 2019). Cells were seeded overnight in a 96-well plate at a concentration of 1 × 10^6^ cells/mL, and then pre-treated with various concentrations of zerumbone (0–50 µM) for 1 h following treatment of LPS (1 μg/ml). After 24 h, cells were incubated with MTT (5 mg/ml) at 37°C for 4 h and then lyzed with acidified isopropanol (0.04 M HCl in absolute isopropanol). Cell viability was measured by determining the absorbance at 570 nm using a microplate reader (BioTek Instruments, Winooski, VT, United States).

### Determination of Nitric Oxide

Nitric oxide (NO) production was measured by determining its stable end product nitrite using a Griess reagent according to the manufacturer’s protocol (Sigma Aldrich, St. Louis, MO, United States). Cells were seeded overnight in a 96-well plate at a concentration of 1 × 10^6^ cells/mL, and then pre-treated with various doses of zerumbone (0–50 µM) for 1 h following treatment of LPS (1 μg/ml). After 24 h, 100 μL cell culture supernatant was harvested and mixed with 100 µL of Griess reagent. Subsequently, the mixture was incubated at room temperature for 10 min and the absorbance at 540 nm was determined using a microplate reader (BioTek Instruments, Winooski, VT, United States). IC_50_ values were calculated from interpolated data using nonlinear regression to fit data to the dose-response curve.

### Enzyme-Linked Immunosorbent Assay

Cells were seeded overnight in a 96-well plate at a concentration of 1 × 10^6^ cells/mL, and then pre-treated with various doses of zerumbone (0–50 µM) for 1 h following treatment of LPS (1 μg/ml). After 24 h, the cell culture supernatant was harvested, and the concentrations of prostaglandin E_2_ (PGE_2_) (Cayman Chemical Company, Ann Arbor, MI, United States), TNF-α, and IL-6 (Thermo Scientific, Waltham, MA, United States) were measured by ELISA according to the manufacturer's protocols. For IL-1β determination, cells were pre-treated with various doses of zerumbone (0–50 µM) for 1 h and then treated with LPS (1 μg/ml) for 5 h following treatment of ATP (5 mM) for 30 min. Cell culture supernatant was harvested and the concentration of IL-1β was measured by ELISA according to the manufacturer's protocols (Thermo Scientific, Waltham, MA, United States). IC_50_ values were calculated from interpolated data using nonlinear regression to fit data to the dose-response curve.

### Western Blotting Analysis

Cells were lyzed by radioimmunoprecipitation assay buffer (RIPA buffer) containing protease inhibitors and phosphatase inhibitors (Sigma Aldrich, St. Louis, MO, United States), and the protein concentration of cell lysate samples was quantified using BCA protein assay (Thermo Scientific, Waltham, MA, United States). Equal amounts of proteins were separated with 8–15% SDS-PAGE and electroblotted onto a PVDF membrane (Millipore, Madrid, Spain). The membrane was incubated with primary antibodies overnight, washed three times by PBS containing 0.05% Tween-20, and then subsequently incubated with horseradish (HRP)-conjugated secondary antibody (Santa Cruz, CA, United States). The immunoreactive bands were detected by ECL chemiluminescence substrate (Thermo Scientific, Waltham, MA, United States). The signals were captured, and the band intensities were quantified using a Bio-Rad ChemiDoc XRS^+^ system (Bio-Rad Laboratories, Inc. Hercules, CA, United States).

### NF-κB Promoter Reporter Assay

J-blue cells are a stably carried NF-κB reporter gene in the J774A.1 cell line, which secrete embryonic alkaline phosphatase (SEAP) induced by NF-κB as described previously (Hung et al., 2019). Cells were seeded overnight in a 96-well plate at a concentration of 1 × 10^6^ cells/mL, and then pre-treated with different concentrations of zerumbone (0–50 µM) for 1 h following treatment of LPS (1 μg/ml). After 24 h, 20 μL cell culture supernatant was harvested, mixed with 200 µL QUANTI-blue medium (InvivoGen, San Diego, CA, United States), and then incubated at 37°C for 45 min. The activity of SEAP was measured by determining the OD at 655 nm using a microplate reader (BioTek Instruments, Winooski, VT, United States).

### Immunofluorescence Staining

Cells were fixed with 4% paraformaldehyde for 15 min and permeabilized with 0.1% Triton-X 100 for 10 min. Cells were incubated with specific primary antibody against ASC (sc-22514-R, Santa Cruz, CA, United States) and caspase-1 (sc-56036, Santa Cruz, CA, United States) overnight, washed with PBS to remove the excessive primary antibodies, and then incubated with fluorescent secondary antibodies. Samples were counterstained with DAPI (Invitrogen, Carlsbad, CA, United States) to visualize the nuclei. After washing, the sections were mounted in VECTASHIELD® mounting medium (Invitrogen, Carlsbad, CA, United States), examined under a confocal laser microscope (Leica, Exton, PA, United States), and analyzed using the Imaris 8 Image Analysis Software (Oxford Instruments, Oxford, United Kingdom).

### Statistical Analysis

All assays were performed in triplicate and data were presented as mean ± standard deviation (SD). Differences between the two groups were evaluated using one-way ANOVA followed by Tukey post-hoc test. The data were analyzed using Graph Pad Prism software version 6.0 for Windows (GraphPad Software, San Diego, CA). A value of *p* < 0.05 was considered as statistically significant.

## Results

### Zerumbone Inhibits the Production of Nitric Oxide and Suppresses the Expression of iNOS and COX-2 in LPS-Activated J774A.1 Cells

**FIGURE 1:**
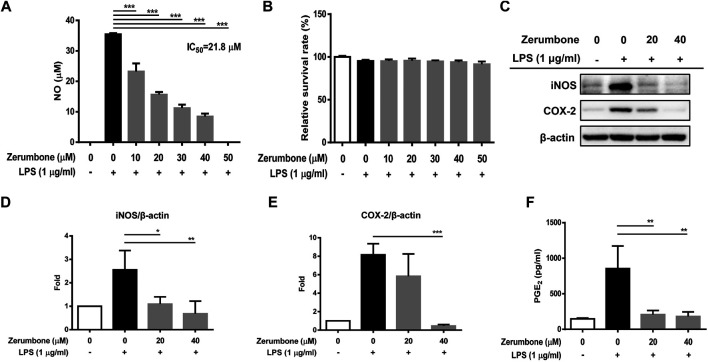
The effects of zerumbone on the NO production, cell viability, and iNOS and COX-2 expressions in LPS-activated J774A.1 cells. Cells were pre-treated with various doses of zerumbone for 1 h following treatment of LPS (1 μg/ml) for 24 h. **(A)** The cell culture supernatant was collected and the production of NO was determined by Griess reagent assay. **(B)** Cell viability was analyzed by MTT assay. **(C)** Cells were harvested and lyzed by lysis buffer, and the expressions of iNOS and COX-2 were determined using Western blot. β-actin was regarded as a loading control. The representative Western blot results were obtained in three separate experiments. **(D–E)** The intensities of bands were quantified from three separate experiments and normalized to untreated samples. **(F)** Cell supernatants were collected and the levels of PGE_2_ were measured by ELISA. Data were represented as means ± SD. Statistical significance between the zerumbone-treated groups vs. the LPS-only group is represented as follows: *p < 0.05, **p < 0.01 and ***p < 0.001.

The product of iNOS catalysis, nitric oxide, plays a key role in mediating many aspects of inflammatory responses (Perez-Sala and Lamas, 2001). To examine whether zerumbone affects the production of nitric oxide by LPS-activated macrophages, J774A.1 cells were pre-treated with various doses (0–50 μM) of zerumbone for 1 h following treatment of LPS (1 μg/ml) for 24 h. The production of nitric oxide was determined by Griess reagent assay. As shown in Figure 1A, LPS treatment induced the expression of NO compared with untreated cells, and zerumbone (10–50 μM) significantly repressed the production of NO in LPS-activated J774A.1 cells. To avoid the inhibitory effects of nitric oxide production in LPS-activated J774A.1 cells due to cell toxicity of zerumbone, we also examined the cell viability using MTT assay. As shown in Figure 1B, no cytotoxic effect was observed when the J774 A.1 cells were treated with zerumbone≦50 µM. Moreover, COX-2 is mainly an inducible isoform that shares significant features with iNOS in regulating inflammatory responses (Perez-Sala and Lamas, 2001). Our experimental results also showed that LPS treatment induced the expression of iNOS and COX-2 compared with untreated cells (Figures 1C–E), and zerumbone significantly suppressed the expression of iNOS (20 and 40 μM) and COX-2 (40 μM) in LPS-activated J774A.1 cells (Figures 1C–E). Moreover, we also found that zerumbone (20 and 40 μM) significantly decreased the production of PGE_2_ by LPS-activated J774A.1 cells (Figure 1F).

### Zerumbone Attenuates the Production of IL-6 in Lipopolysaccharide-Activated J774 A.1 Cells

**FIGURE 2:**
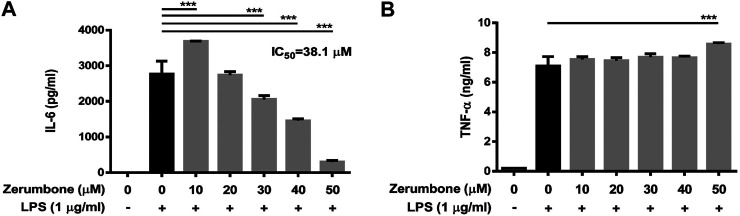
The effects of zerumbone on the production of IL-6 and TNF-α in LPS-activated J774A.1 cells. Cells were pre-treated with various doses of zerumbone for 1 h following treatment of LPS (1 μg/ml) for 24 h. Cell supernatants were collected and the levels of **(A)** IL-6 and **(B)** TNF-α were analyzed using ELISA. Data were collected in triplicate and represented as means ± SD. Statistical significance between the zerumbone-treated groups vs. the LPS-only group is represented as follows: ***p < 0.001.

To examine whether zerumbone affects the production of proinflammatory cytokines by LPS-activated J774 A.1 cells, the cells were pre-treated with various doses (0–50 μM) of zerumbone for 1 h following treatment of LPS (1 μg/ml) for 24 h. The cell culture supernatants were collected and the production of IL-6 and TNF-α was determined by ELISA. As shown in Figure 2A, LPS treatment induced the production of TNF-α and IL-6 compared with untreated cells (Figures 2A,B). Low dose (10 μM) of zerumbone significantly increased production of IL-6 by LPS-activated J774A.1 cells, whereas higher doses (20–50 μM) of zerumbone significantly suppressed production of IL-6 by LPS-activated J774A.1 cells in a dose-dependent manner (Figure 2A). For the production of TNF-α, zerumbone did not affect the secretion of TNF-α at low doses (10–40 μM) but significantly increased the secretion of TNF-α at high dosage (50 μM) of zerumbone by LPS-activated J774A.1 cells (Figure 2B).

### Zerumbone Inhibits the Phosphorylation of ERK and Decreases the Activity of NF-κB in Lipopolysaccharide-Activated J774.1 Cells

**FIGURE 3:**
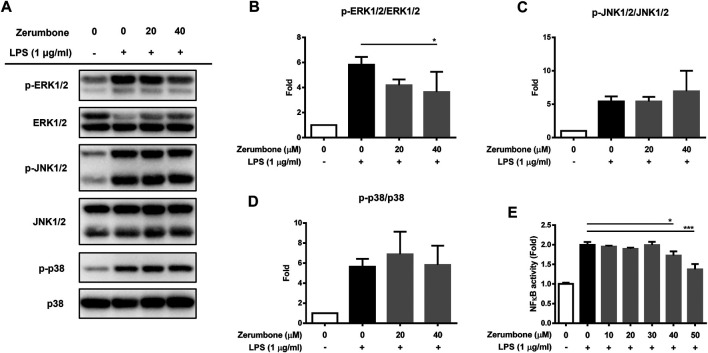
The effects of zerumbone on the activation of MAPK and NF-κB in LPS-activated J774A.1 cells. Cells were pre-treated with various doses of zerumbone for 1 h following treatment of LPS (1 μg/ml) for 2 h. **(A)** The expressions of MAPK-associated proteins were determined by Western blot. Phospho-specific signals were then normalized against the total level of the target protein, using that protein as its own internal loading control for maximum accuracy. The representative Western blot results were obtained in three separate experiments. **(B–D)** The intensities of bands were quantified from three separate experiments and normalized to untreated samples. Data were resented as means ± SD. **(D)** J-blue cells, a stable NF-κB reporter cell line generated by transfected J774A.1 cells with NF-κB SEAP report plasmids, were pre-treated with various doses of zerumbone for 1 h following treatment of LPS (1 μg/ml) for 6 h. The activation of NF-κB was examined by SEAP activity. Data were represented as means ± SD. Statistical significance between the zerumbone-treated groups vs. the LPS-only group is represented as follows: *p < 0.05, **p < 0.01 and ***p < 0.001.

The MAPK/NF-κB signaling pathway plays a crucial role in modulating the development and progression of inflammatory responses (Hung et al., 2019). To examine whether zerumbone regulates the activation of MAPK signaling pathway, by LPS-activated J774 A.1 cells, cells were pre-treated with various doses (20 and 40 μM) of zerumbone for 1 h following treatment of LPS (1 μg/ml) for 2 h. The expression levels of MAPK were analyzed by Western blot. Our experimental results showed that LPS treatment induced the phosphorylation of ERK, JNK, and p38 MAPK compared with untreated cells (Figures 3A–D), while zerumbone significantly inhibited the phosphorylation of ERK (at the concentration of 40 µM zerumbone) by LPS-activated J774A.1 cells, but did not affect the phosphorylation of JNK and p38 MAPK (Figures 3A–D). Additionally, the effect of zerumbone on the activity of NF-κB in macrophages was also examined. J-blue cells, a stable NF-κB reporter cell line generated by transfected J774A.1 cells with NF-κB secrete embryonic alkaline phosphatase (SEAP) reported plasmids, which induced the secretion of SEAP by NF-κB activation (Hung et al., 2019). The J-blue cells were pre-treated with various doses of zerumbone for 1 h following treatment of LPS (1 μg/ml) for 6 h, then the activation of NF-κB was examined by SEAP activity. Our results showed that LPS treatment induced the activation of NF-κB compared with untreated cells, and high dosages (40 and 50 μM) of zerumbone significantly attenuated the activity of NF-κB (Figure 3E).

### Zerumbone Suppresses the Production of IL-1β and Inhibits the Activity of the NLRP3 Inflammasome in Lipopolysaccharide/ATP- and LPS/Nigericin-Activated J774A.1 Cells

**FIGURE 4:**
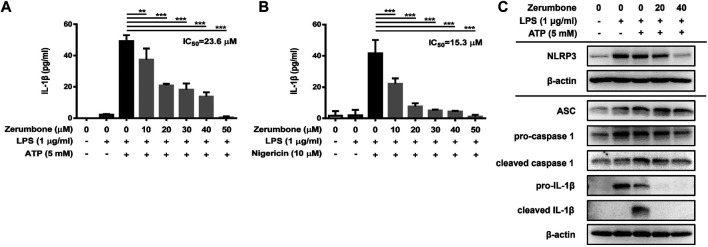
The effects of zerumbone on the production of IL-1β and the activity of NLRP3 inflammasome in LPS/ATP- and LPS/nigericin-activated J774A.1 cells. Cells were pre-treated with various doses of zerumbone for 1 h and then treated with LPS (1 μg/ml) for 5 h following treatment of ATP (5 mM) or nigericin (10 μΜ) for 30 min. **(A, B)** Cell culture supernatants were collected and the levels of IL-1β were determined by ELISA. Data were represented as means ± SD. Statistical significance between the zerumbone-treated groups vs. the LPS/ATP or LPS/nigericin groups is represented as follows: *p < 0.05, **p < 0.01 and ***p < 0.001. **(C)** The expression levels of inflammasome-associated proteins were analyzed by Western blot. β-actin was regarded as a loading control. The representative Western blot results were obtained in three separate experiments.

**FIGURE 5:**
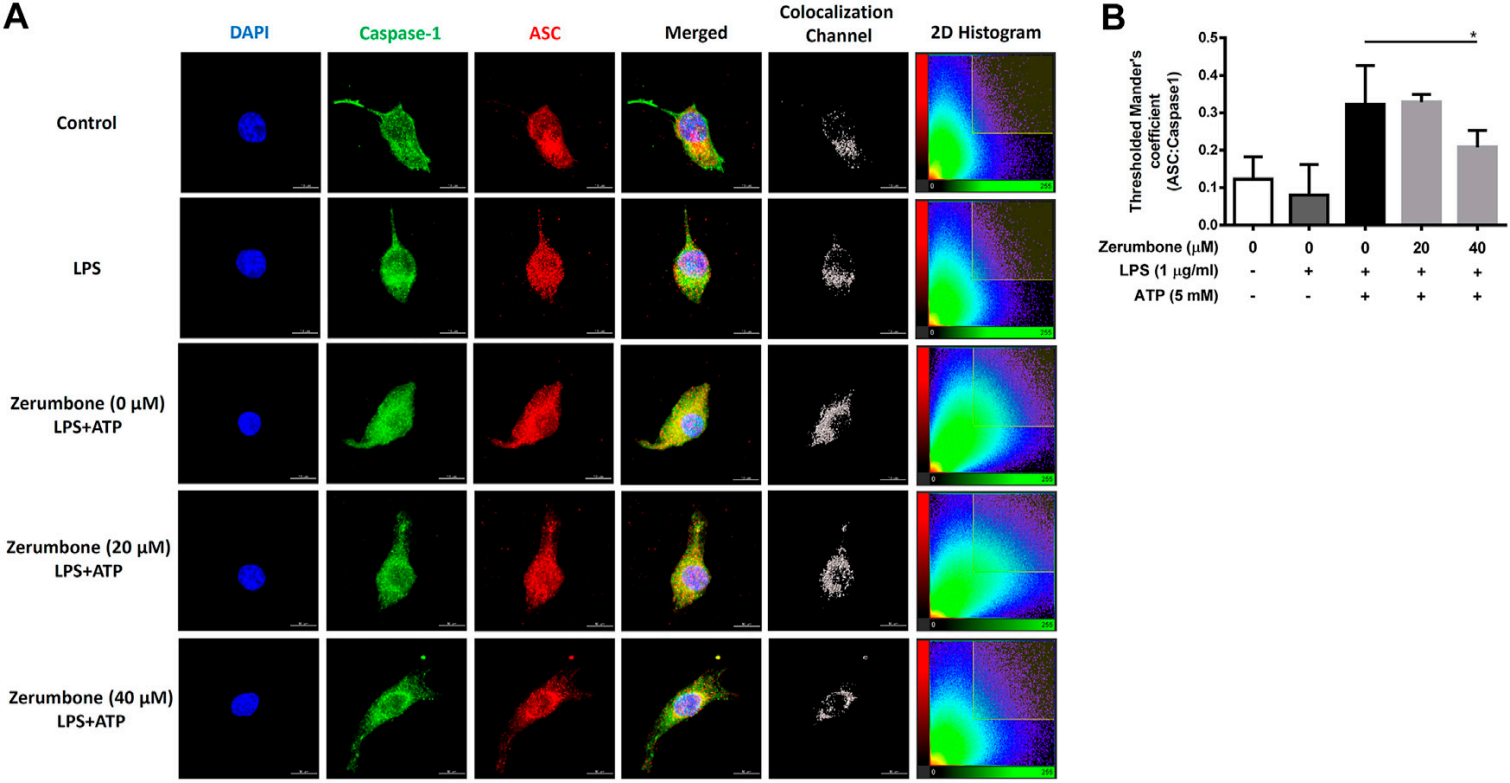
The effects of zerumbone on the colocalization of NLRP3 inflammasome components (ASC and caspase-1) in LPS/ATP-activated J774 A.1 cells. Cells were pre-treated with various doses of zerumbone for 1 h and then treated with LPS (1 μg/ml) for 5 h following treatment of ATP (5 mM) for 30 min. **(A)** Cells were stained with caspase-1 (green), ASC (red), and DAPI (blue) and then analyzed using confocal microscopy and Imaris software for confocal image 3D reconstruction (B) ASC speck formation was analyzed by the threshold of 2D histogram in panel A to determine the colocalization of caspase-1 and ASC signals using Mander’s coefficient. Data were represented as means ± SD. Statistical significance between the zerumbone-treated groups vs. the LPS/ATP group is represented as follows: *p < 0.05.

The activation of NLRP3 inflammasome plays an important role in modulating the secretion of IL-1β, a critical regulator of the inflammatory response (Hung et al., 2019). To investigate whether zerumbone regulates the activation of NLRP3 inflammasome, J774A.1 cells were pre-treated with various doses (0–50 μM) of zerumbone for 1 h and then treated with LPS (1 μg/ml) for 5 h following treatment of ATP (5 mM) or nigericin (10 μM) for 30 min. Cell culture supernatants were harvested and the levels of IL-1β were measured by ELISA, while the expression levels of inflammasome-associated proteins were analyzed by Western blot. As shown in Figures 4A,B, LPS/ATP and LPS/nigericin treatments induced the secretion of IL-1β compared with untreated cells, and zerumbone significantly repressed the secretion of IL-1β by LPS/ATP- and LPS/nigericin-activated J774A.1 cells in a dose-dependent manner. Moreover, our experimental results showed that LPS/ATP treatment induced the expression of NLRP3, ASC, pro-caspase-1, cleaved caspase-1, pro-IL-1β and cleaved IL-1β compared with untreated cells, and zerumbone inhibited the expression of NLRP3, pro-caspase-1, cleaved caspase-1, pro-IL-1β and cleaved IL-1β in LPS/ATP-activated J774A.1 cells (Figure 4C). Furthermore, the colocalization of NLRP3 inflammasome components (ASC and caspase-1) was also examined since it has been considered as a marker for assessing inflammasome activation (Zhang et al., 2012). Our experimental results indicated that LPS/ATP treatment promoted the colocalization of ASC and caspase-1 compared with untreated cells, indicating that LPS/ATP treatment induced the activation of the NLRP3 inflammasome by J774.1 cells. And higher doses (40 μM) of zerumbone decreased the colocalization of ASC and caspase-1 in LPS/ATP-activated J774 A.1 cells (Figures 5A,B).

### Zerumbone Represses the Production of Nitric Oxide and Proinflammatory Cytokines in Lipopolysaccharide-Activated Murine Peritoneal Macrophages and Bone Marrow-Derived Macrophages

**FIGURE 6:**
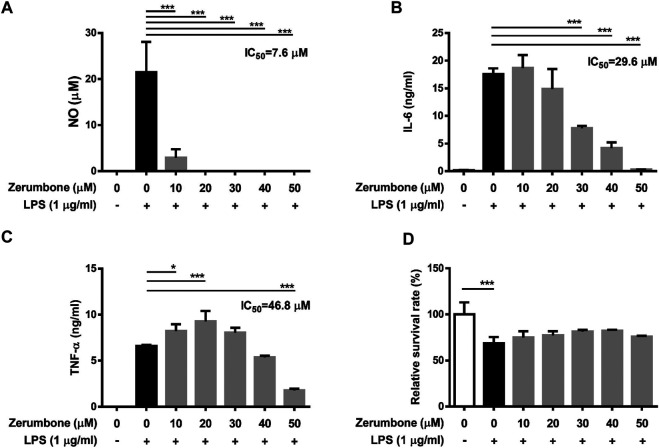
The effects of zerumbone on the NO, IL-6, and TNF-α productions and cell viability in LPS-activated murine peritoneal macrophages. Cells were pre-treated with various doses of zerumbone for 1 h following treatment of LPS (1 μg/ml) for 24 h. **(A)** The production of NO was determined by Griess reagent assay. The expression levels of **(B)** IL-6 and **(C)** TNF-α were measured using ELISA. Statistical significance between the zerumbone-treated groups vs. the LPS-only group is represented as follows: *p < 0.05 and ***p < 0.001. **(D)** Cell viability was analyzed by MTT assay. Data were represented as means ± SD. Statistical significance is represented as follows: ***p < 0.001, compared with the LPS-only group.

**FIGURE 7:**
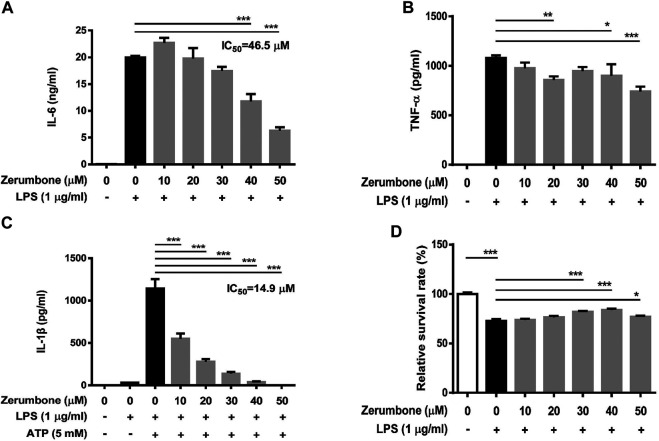
The effects of zerumbone on the cell viability and IL-6, TNF-α, and IL-1β productions in LPS- or LPS/ATP-activated murine bone marrow-derived macrophages. Cells were pre-treated with various doses of zerumbone for 1 h following treatment of LPS (1 μg/ml) for 24 h. The expression levels of **(A)** IL-6 and **(B)** TNF-α were measured using ELISA. Statistical significance between the zerumbone-treated groups vs. the LPS-only group is represented as follows: *p < 0.05, **p < 0.01 and ***p < 0.001. **(C)** For the IL-1β secretion, cells were pre-treated with various doses of zerumbone for 1 h and then treated with LPS (1 μg/ml) for 5 h following treatment of ATP (5 mM) for 30 min. Cell culture supernatants were collected and the levels of IL-1β were determined by ELISA. Data were represented as means ± SD. Statistical significance between the zerumbone-treated groups vs. the LPS-only group is represented as follows: ***p < 0.001. **(D)** Cell viability was analyzed by MTT assay. Statistical significance between the zerumbone-treated groups vs. the LPS-only group is represented as follows: *p < 0.05 and ***p < 0.001.

To further confirm the anti-inflammatory effects of zerumbone, murine peritoneal macrophages were isolated and pre-treated with various doses (0–50 μM) of zerumbone for 1 h following treatment of LPS (1 μg/ml) for 24 h. The production of NO and proinflammatory cytokines (IL-6 and TNF-α) were determined by Griess reagent assay and ELISA respectively. Our experimental results showed that LPS treatment induced the production of NO, IL-6, and TNF-α compared with untreated cells (Figures 6A–C), and zerumbone significantly repressed the production of NO (10–50 μM) and IL-6 (30–50 μM) in LPS-activated murine peritoneal macrophages (Figures 6A,B). For the effect of zerumbone on TNF-α production in peritoneal macrophages, our experimental results showed that zerumbone had a different effect on TNF-α production depending on the concentration utilized (increased at 10–20 μM but decreased at 50 μM). Although LPS treatment decreased the cell survival compared with untreated cells, no cytotoxic effects were observed when cells were treated with these doses of zerumbone (Figure 6D). Additionally, the anti-inflammatory effects of zerumbone in bone marrow-derived macrophages were also examined. Our experimental results showed that LPS or LPS/ATP treatments induced the production of IL-6, TNF-α, and IL-1β compared with untreated cells (Figures 7A–C), while zerumbone significantly suppressed the production of IL-6 (40–50 μM) and TNF-α (20 and 40–50 μM) in LPS-activated murine bone marrow-derived macrophages (Figures 7A,B). Moreover, zerumbone also inhibited the production of IL-1β (10–50 μM) in LPS/ATP-activated murine bone marrow-derived macrophages (Figure 7C). In addition, our experimental results showed that LPS treatment decreased the cell survival of murine bone marrow-derived macrophages compared with untreated cells; however, zerumbone (20–50 μM) increased cell survival of murine bone marrow-derived macrophages compared with LPS alone (Figure 7D).

## Discussion

Inflammation is a biological response of the immune system to harmful stimuli such as pathogens and injury, and is a protective response involving immune cells and molecular mediators (Chen et al., 2018); however, an overactive inflammatory response can trigger a cytokine storm, a situation in which excessive or uncontrolled release of proinflammatory cytokines can damage organs and eventually lead to the death of the organism (D'Elia et al., 2013). Macrophages are extremely important immune cells involved in the host defense mechanism during the inflammatory response, which secrete various inflammatory mediators such as NO and proinflammatory cytokines, TNF-α, IL-6, and IL-1β during activation (Hung et al., 2019). Well-controlled inflammatory responses have benefits in disease progression and mortality.

LPS is a pivotal inducer of inflammatory processes, which promotes the inflammatory responses in macrophages by the production of inflammatory mediators, including TNF-α, IL-6, iNOS, and COX-2. Therefore, three kinds of macrophages, murine J774A.1 macrophage cell line, murine peritoneal macrophages, and murine bone marrow-derived macrophages were used and *in vitro* and *ex vivo* experiments in the present study were performed. Our experimental results demonstrated that LPS-activated macrophages strongly induced the production of NO, IL-6, and TNF-α and promoted the expression of iNOS and COX-2, whereas zerumbone treatment exhibited an overall anti-inflammatory effect through suppressing LPS-induced NO, IL-6, PGE_2_, and TNF-α (a weak inhibitory effect) production as well as iNOS and COX-2 expressions in macrophages, except for the production of TNF-α in J774A.1 cells (inhibition of ERK in J774A.1 cells somehow increases the production of TNF-α (Kim et al., 2004)). In addition, compared to the induction levels of TNF-α among these cells, LPS 1 μg/ml primed the higher TNF-α production in J774A.1 cells than murine peritoneal macrophages and bone marrow-derived macrophages. It is possible that the inhibitory effect of zerumbone on the production of TNF-α is relatively weak in an excessive inflammatory stimulation. A previous study also indicated that zerumbone inhibits the expression of TNF-α, IL-1β, PGE_2_, and COX-2 proteins in LPS-induced human U937 macrophages [9]. Based on these findings, zerumbone exerts anti-inflammatory effect by inhibiting the production of inflammatory mediators in LPS-treated macrophages.

The stimulation of TLR4 by LPS triggers the TLR-stimulated MAPK signaling which involves interesting crosstalk with the IKK/NF-κB pathway (Shi and Sun, 2018); therefore, activation of MAPK and NF-κB pathways are common inflammatory signaling pathways that can lead to proinflammatory cytokine production (Xiao et al., 2020). In the present study, we found that zerumbone significantly inhibited the phosphorylation of ERK by LPS-activated J774A.1 cells but did not affect the phosphorylation of JNK and p38 MAPK. ERK-MAPK signaling is crucial for the production of NO (Kim et al., 2004) and the secretion of IL-6 and TNF-α (Olsnes et al., 2011). Our results suggest that zerumbone inhibits the production of NO and the secretion of IL-6 and TNF-α through blocking ERK-MAPK signaling. A previous study indicated that mitogen-activated protein 3 kinase Tpl2 is required for ERK-MAPK activation in murine bone marrow-derived macrophages with LPS stimulation (Banerjee et al., 2006). Whether zerumbone selectively inhibited ERK through attenuating the phosphorylation of Tpl2 might need further examination. On the other hand, the transcription factor NF-κB plays an important role in administrating the transcription of a variety of cellular genes regulating the inflammatory response (Dorrington and Fraser, 2019). Our experimental results demonstrated that zerumbone attenuates the activity of NF-κB in J-blue cells. Haque *et al.* also indicated that zerumbone significantly downregulated the phosphorylation of NF-κB (p65), IκBα, and IKKα/β as well as restoring the degradation of IκBα in human U937 macrophages (Haque et al., 2018). Examination of NF-κB signaling dynamics that translocate into the nucleus is critical in determining the quality and quantity of the inflammatory response in pathogen-challenged macrophages (Dorrington and Fraser, 2019). Hwang et al. demonstrated that zerumbone suppressed enterotoxigenic *bacteroides fragilis* infection-induced colonic inflammation through inhibition of NF-κB activation and nuclear translocation (Hwang et al., 2019). In addition, Lee *et al.* indicated that zerumbone ameliorated lipopolysaccharide-induced cytokine expression via the p38 MAPK/JNK-IκB/NF-κB pathway in a murine acute lung injury model (Lee et al., 2018). Collectively, these results suggest that zerumbone suppresses the LPS-induced inflammatory response via both ERK-MAPK and NF-κB signaling pathways in macrophages. In other types of cells, zerumbone also has potential in inhibiting the NF-κB signaling pathway in gastric cancer cells (Tsuboi et al., 2014) and pancreatic cancer cells (Tsuboi et al., 2014), resulting in the blocking of angiogenesis.

The NLRP3 inflammasome plays a critical role in inflammatory response as a major component of innate immunity and is considered a promising target for the treatment of many diseases associated with inflammation (Christgen et al., 2020). Our experimental results demonstrated that zerumbone inhibited the expression of NLRP3, pro-caspase-1, cleaved caspase-1, pro-IL-1β and cleaved IL-1β, and attenuated the secretion of IL-1β in LPS/ATP-activated J774A.1 cells as well as suppressing the secretion of IL-1β in LPS/nigericin-activated J774A.1 cells. Moreover, our experimental results also showed that zerumbone decreased the colocalization of ASC and caspase-1 in LPS/ATP-activated J774A.1 cells, indicating that zerumbone inhibits the formation of NLRP3 inflammasome. Collectively, these results demonstrate that zerumbone decreases the production of IL-1β by blocking the activation of the NLRP3 inflammasome. Inhibition of IL-1β secretion by zerumbone has also been reported in streptozotocin-induced diabetic nephropathy and LPS-induced acute lung injury animal models (Tzeng et al., 2013; Lee et al., 2018). On the other hand, the activation of NLRP3 inflammasome and the expression of IL-1β are associated with cancer development, metastasis, and poor prognosis in several types of cancers, including breast (Jeon et al., 2016), lung (Wang et al., 2016), colorectal (Deng et al., 2019), and glioma (Yin et al., 2018), etc. Previous studies showed that zerumbone inhibits the expression of IL-β and suppresses IL-1β-induced cell migration and invasion in triple-negative breast cancer (Han et al., 2014; Jeon et al., 2016). This evidence implies that zerumbone exerts anti-inflammatory and anti-cancer activities by attenuating the activation of NLRP3 inflammasome.

Evidence from *in vivo* experimentations has also demonstrated anti-inflammatory effects of zerumbone. Murakam et al. pointed out that oral administration of zerumbone significantly suppresses the levels of IL-1β, TNF-α and PGE_2_ in a dextran sodium sulfate-induced colitis mouse model (Murakami et al., 2003). Sulaiman *et al.* indicated that intraperitoneal administration of zerumbone inhibits paw edema in a carrageenan-induced acute inflammatory model in mice (Sulaiman et al., 2010). In addition, zerumbone also attenuates chronic inflammation by suppressing granulomatous tissue formation in a cotton pellet-induced granuloma mouse model (Sulaiman et al., 2010). As similar to our findings, Ho *et al.* demonstrated that zerumbone inhibits TNF-α and IL-6 productions, decreases COX-2 and iNOS expressions and suppresses NF-κB activation in an LPS-induced acute lung injury mouse model (Ho et al., 2017). Moreover, Lee *et al.* indicated that zerumbone attenuates IL-1β production, inhibits MAPKs phosphorylation, decreases NF-κB activation, suppresses neutrophils infiltration, and inhibits the expression of adhesion molecules intercellular adhesion molecule-1 (ICAM-1) and vascular cell adhesion molecule-1 (VCAM-1) in an LPS-induced acute lung injury mouse model (Lee et al., 2018).

**FIGURE 8:**
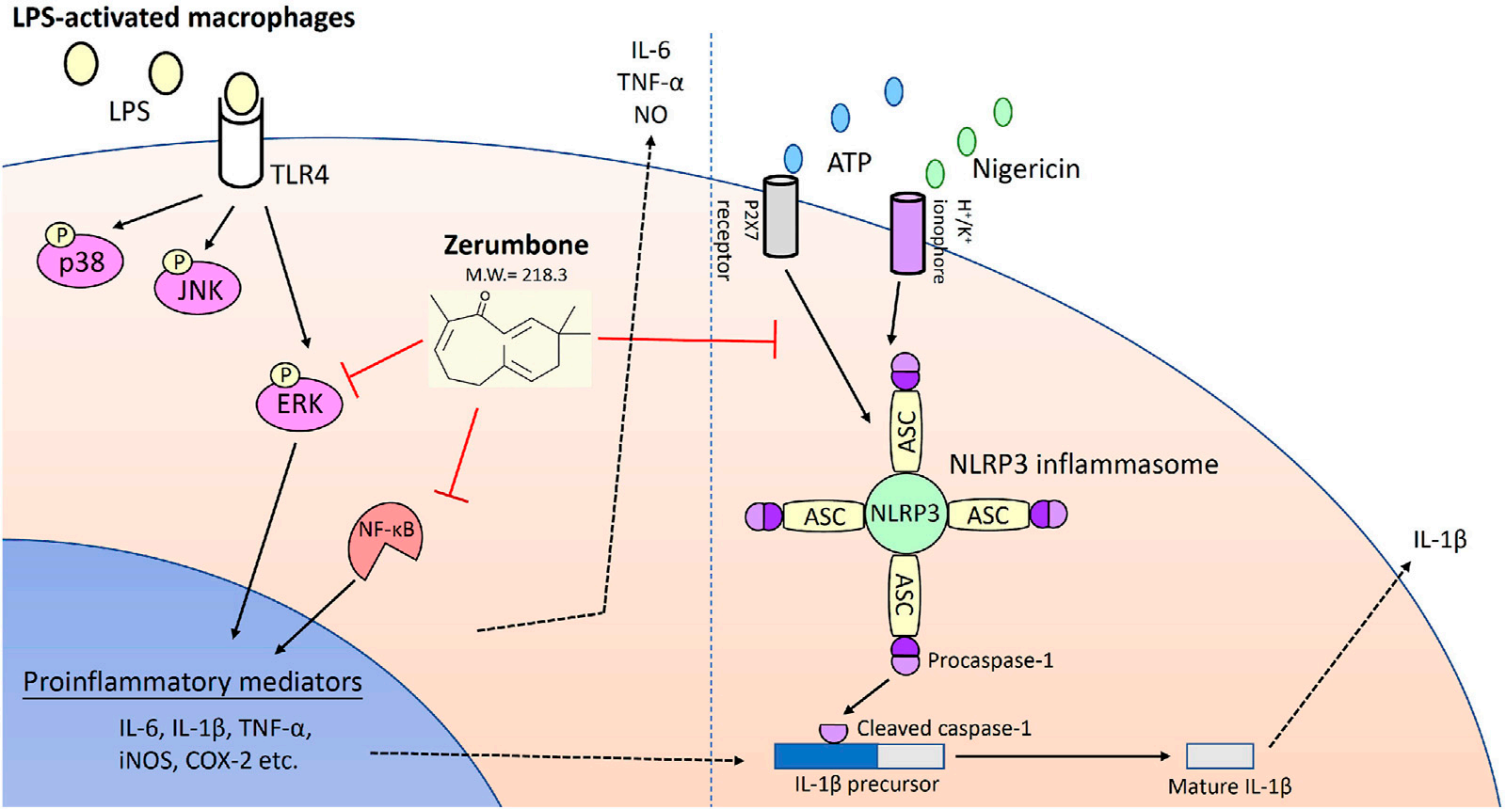
Zerumbone suppresses the LPS-induced inflammatory response and represses activation of the NLRP3 inflammasome in macrophages. Zerumbone effectively attenuates LPS-induced inflammatory responses in macrophages both *in vitro* and *ex vivo* by suppressing activation of the ERK-MAPK and NF-κB signaling pathways, inhibiting LPS-induced inflammatory mediators, and blocking the activation of the NLRP3 inflammasome.

In conclusion, we demonstrated that zerumbone, a main component isolated from *Zingiber zerumbet* (L.) Smith, could effectively attenuate LPS-induced inflammatory response both *in vitro* and *ex vivo* experiments of the macrophages used in this study through suppressing the activation of both ERK-MAPK and NF-κB signaling pathways as well as blocking the activation of NLRP3 inflammasome (Figure 8). These results suggest zerumbone could be an effective treatment for sepsis and inflammasome-related diseases.

## Data Availability

The raw data supporting the conclusions of this article will be made available by the authors, without undue reservation.
